# Additively Manufactured Partially Porous PAEK Topologies for Proximal Tibia Revision Cones and Sleeves

**DOI:** 10.1007/s10439-026-04042-8

**Published:** 2026-02-19

**Authors:** Paul M. DeSantis, Emma Barnes, Abigail Tetteh, James A. Smith, Drew Mike, Corey Perine, Steven M. Kurtz

**Affiliations:** 1 Implant Research Core, School of Biomedical Engineering, Science and Health Systems, Drexel University, Philadelphia, PA 19104 USA; 2 Maxx Orthopedics, Inc., Norristown, PA 19403 USA

**Keywords:** Additive manufacturing, 3D printing, Polyaryletherketone, PAEK, PEEK, Metaphyseal cone, Metaphyseal sleeve

## Abstract

Metaphyseal cones and sleeves are an increasingly common component utilized in revision total knee arthroplasty. These partially porous devices have a high surface area and aim to integrate with cancellous bone of the distal femur and proximal tibia to increase the stability of the implant. Incidences of metal-hypersensitivity and macrophage-stimulated osteolysis have motivated the development of a non-metal alternative in the form of   polyaryletherketone (PAEK) implants. We hypothesized that highly complex porous PAEK components could be additively manufactured to withstand the mechanical forces of the knee, while still allowing for bone ingrowth. Taguchi optimization was used to investigate parameters and porous geometries that achieved the highest ultimate load in 45° shear-compression. Micro-CT was used to evaluate the quality of the print porosity for each experimental group. Low-melt (LM) PAEK sample means exceeded all polyetheretherketone (PEEK) means, and geometry and percent porosity were found to be the most significant parameters for LM PAEK, while nozzle temperature, chamber temperature and layer height were the most significant for PEEK (*p* < 0.05). All samples passed the theoretical worst-case scenario of ~ 5.5 kN ultimate load in 45° shear. The mean errors for percent porosity were not significantly different (*p* > 0.05) and measured to be − 8.46% and − 12.25% for PEEK and LM PAEK groups, respectively. This work serves as a proof-of-concept for 3D printing highly porous non-metal metaphyseal sleeves/cones without compromising on strength.

**Supplementary Information:**

The online version contains supplementary material available at 10.1007/s10439-026-04042-8.

## Introduction

Total knee arthroplasty (TKA) is an effective treatment for patients suffering from end-stage osteoarthritis, and approximately 90% of implants survive between 10 and 15 years [1]. In cases where the implant fails, a revision TKA is performed, however these revision implants are also capable of failure. In a meta-analysis of 39,723 revision TKAs performed between 2014 and 2024, 13.5% required re-revision. The most common reasons for revision failure were periprosthetic joint infection (27.5%), instability (13.6%), aseptic loosening (12.8%), arthrofibrosis (9.4%), unexplained pain (8.0%), and periprosthetic fracture (7.6%) [2].

A major challenge in revision TKA is the management of bone loss. The variance in the size and location of bone defects and the quality of remaining bone make it difficult to achieve a stable implant fixation [3]. While bone cement can be used to achieve a union of the revised implant and native bone, an increasingly favorable alternative to bone cement is the use of metaphyseal sleeves and cones [4]. These porous, high surface area implants aim to provide mechanical stability, while also allowing for integration with the cancellous bone of the distal femur and proximal tibia via bone ingrowth [5].

The Anderson Orthopaedic Research Institute (AORI) scale classifies femoral and tibial defects into types I, II, and III. The most severe types of defect are type IIb—where both femoral/tibial condyles experience metaphyseal bone damage and cancellous bone loss—and type III—where there is a major defect in the metaphyseal bone requiring a complete structural replacement [6]. Both sleeves and cones have been shown to effectively manage type IIb and III defects [3, 4, 7]. No differences in overall implant survival or clinical outcome in short- nor long-term follow-ups have been detected between cone or sleeve use [8].

These revision constructs, with encouraging survivorship in the absence of infection, are entirely metal components which introduce different challenges to TKA [9–11]. While both sleeves and cones are frequently associated with periprosthetic joint infections (PJI), cones have been associated with infection rates of PJI as high as 11.7%, marking this as a source of concern [4, 8, 11, 12]. Metal sleeves are prone to corrosion-induced complications like metal-protein complex hypersensitivity and wear-induced complications like local macrophage-stimulated osteolysis [13–17]. In cases of well-functioning implants, metal sensitivity may be present in as high as 1 in 4 patients [18]. For failed, loose, or poorly functioning implants, metal sensitivity incidence reportedly increases to 3 in 5 patients [18]. Osteolysis was found to be more prevalent in cementless TKA (6–30%) than cemented TKA (0–16%), introducing an additional complication for non-cemented components [19].

Recent alternatives to metal in orthopedics include a family of thermoplastics called polyaryletherketones (PAEKs). Valued for their biological, mechanical, and chemical properties, PAEKs were introduced to orthopedics as a result of the growing need for materials with a modulus similar to bone [20]. Recently, polyetheretherketone (PEEK)’s utility in 3D printing has been instrumental in the material’s advancement for more complex and personalized biomedical applications [21, 22]. Low-melt polyaryletherketone (LM PAEK) was also introduced in response to PEEK’s high thermal demand, with the goal to reduce bonding time at a lower viscosity, ideally producing parts at a quicker rate [23–25].

The integration of the bone matrix with a 3D printed device is critical to its success in the context of arthroplasty. Techniques like sintering, meshes, and coatings have been used to roughen implant surfaces to achieve this [26, 27]. Uniquely fabricated with additive manufacturing, implants with unit-cell porous interfaces are better able to mimic cancellous bone and integrate more solidly than those with a smooth surface. There are many factors that have yet to be optimized to enhance this osseointegration, with a focus on material, pore size, unit-cell morphology, and percent porosity [28–31].

In this study, we sought to answer the following questions: (1) Which printing and geometric parameters have the greatest influence on the mechanical performance of semi-porous cylinders printed in PEEK and LM PAEK? (2) Which parameter values yield the highest ultimate load for both materials? (3) How accurate is the porosity of the printed specimens to the expected values?

## Materials and Methods

### Taguchi Design of Experiments

The Taguchi method allows for the creation of a design of experiments that modulates multiple parameters without compromising specimen quality [32]. In this case, the control factors (inputs) were design parameters for a 3D printed cylinder, and the outputs were the results of mechanical testing. For this experiment, ultimate load in 45° shear was chosen as the relevant performance characteristic. The purpose of the Taguchi optimization was to determine which parameters tested had the most significant impact on the mechanical performance of the PAEKs, as well as which specific values produced the strongest parts. This data can be interpreted to determine the optimal print parameters for each part based on the material chosen.

The Taguchi optimization process utilizes a loss function that must be minimized and is evaluated by calculating the signal-to-noise ratio (SNR). The SNR is evaluated based on the experimental results and shows which parameters most reduce variability. Greater SNR values also indicate that the parameter has a larger impact on the performance characteristic. Depending on the scenario of the experiment, one may aim to maximize, minimize, or set the performance characteristic to a specific value. In evaluating the mechanical properties of 3D printed parts, the goal was to maximize the performance characteristic of ultimate load, therefore the following SNR equation was utilized:

1 $$SN_{i} = - 10\log \left[ {\frac{1}{{N_{i} }}\mathop \sum \limits_{u = 1}^{{N_{i} }} \frac{1}{{y_{u}^{2} }}} \right]$$

Where *i* represents the experiment number, *u* is the trial number, *N*_*i*_ is the number of trials for experiment *i*, and *y* is the performance characteristic (ultimate load). The SNR is calculated for each experiment, and the average SNR value is calculated for each input factor and level.

Five parameters were chosen to be evaluated based on previous work from our group [32–34] as well as what was reported in the literature [35, 36]. The first three parameters: nozzle temperature, chamber temperature, and layer height are known to influence layer-by-layer adhesion and the crystallization kinetics of the FFF process, subsequently influencing mechanical properties [37, 38]. The last two parameters: geometry type and percent porosity, influenced the properties of the porous region of the cylinder. Increased porosity can improve the amount of bone ingrowth, but at the expense of decreased mechanical strength [34, 39].

The levels for the parameters of nozzle temperature, chamber temperature, and layer height were chosen based on recommendations from the filament manufacturers and what was reported in the literature [32, 37, 40]. For geometry type, gyroid and diamond lattices were chosen as they are two of the most widely used triply periodic minimal surfaces (TPMS) for additive manufacturing, with gyroid closely resembling the natural structure of trabecular bone and diamond being known for its relatively simple repeating structure but large load carrying-capacity [41]. Finally, porosity values of 50 and 70% were chosen as they have previously been shown to improve bone ingrowth [31, 42, 43]. The varied parameters along with constant parameters for the 3D printed PAEK samples are shown in Table 1. Preliminary testing was conducted to confirm that the parameters chosen produced parts that could be printed to completion using the available equipment.

**Table 1:** Print parameters for 3D printing process, including the two levels for varied parameters (L1 and L2)

Constant parameters		Varied parameters		L1	L2
Bed temperature (°C)	250	P1	Nozzle temp. (°C)	380	400
Speed (mm/min)	1500	P2	Chamber temp. (°C)	200	230
Extrusion width (mm)	0.4	P3	Layer height (mm)	0.2	0.3
Extrusion mult., solid region (%)	0.96	P4	Geometry type	Gyroid	Diamond
Extrusion mult., porous region (%)	0.75	P5	Porosity (%)	50	70
Perimeter overlap (mm)	0.5				

Selecting five parameters with two levels corresponds to a Taguchi L8 array, with eight experiments defined using Minitab 22.1 (Minitab LLC, State College, PA) as shown in Table 2.

**Table 2:** Orthogonal array for Taguchi design of experiments (L8, *n* = 4)

Experiment #	P1	P2	P3	P4	P5
X1	380	200	0.2	Gyroid	50
X2	380	200	0.2	Diamond	70
X3	380	230	0.3	Gyroid	50
X4	380	230	0.3	Diamond	70
X5	410	200	0.3	Gyroid	70
X6	410	200	0.3	Diamond	50
X7	410	230	0.2	Gyroid	70
X8	410	230	0.2	Diamond	50

### Design of Specimens

**Fig. 1:**
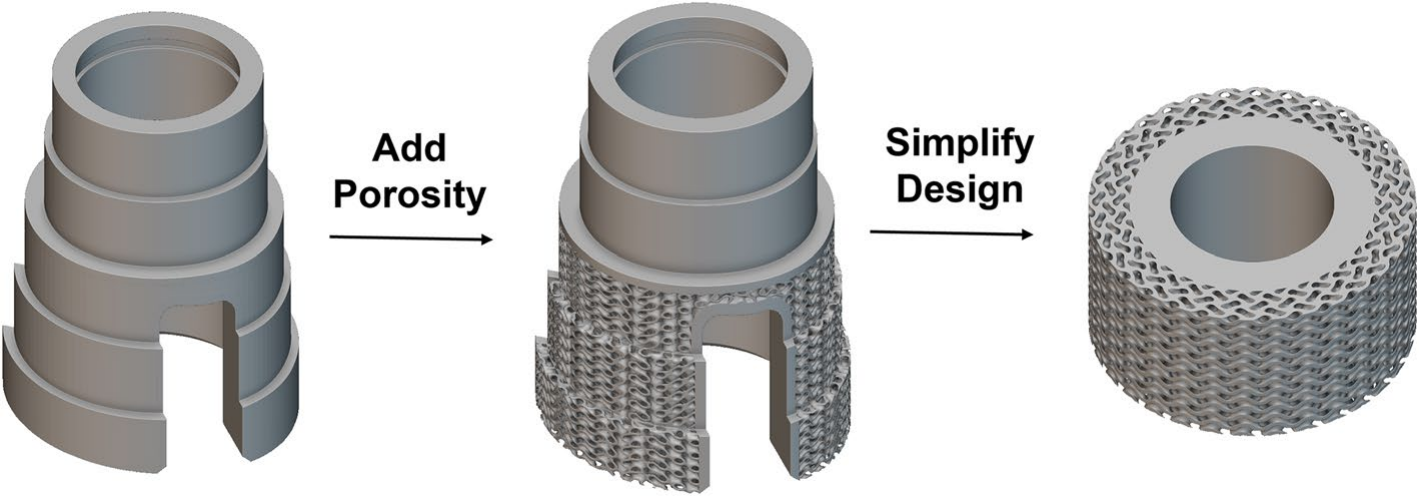
A visualization of the progression of the design process from a metaphyseal cone to a porous cone to a representative simplified porous cylinder

**Fig. 2:**
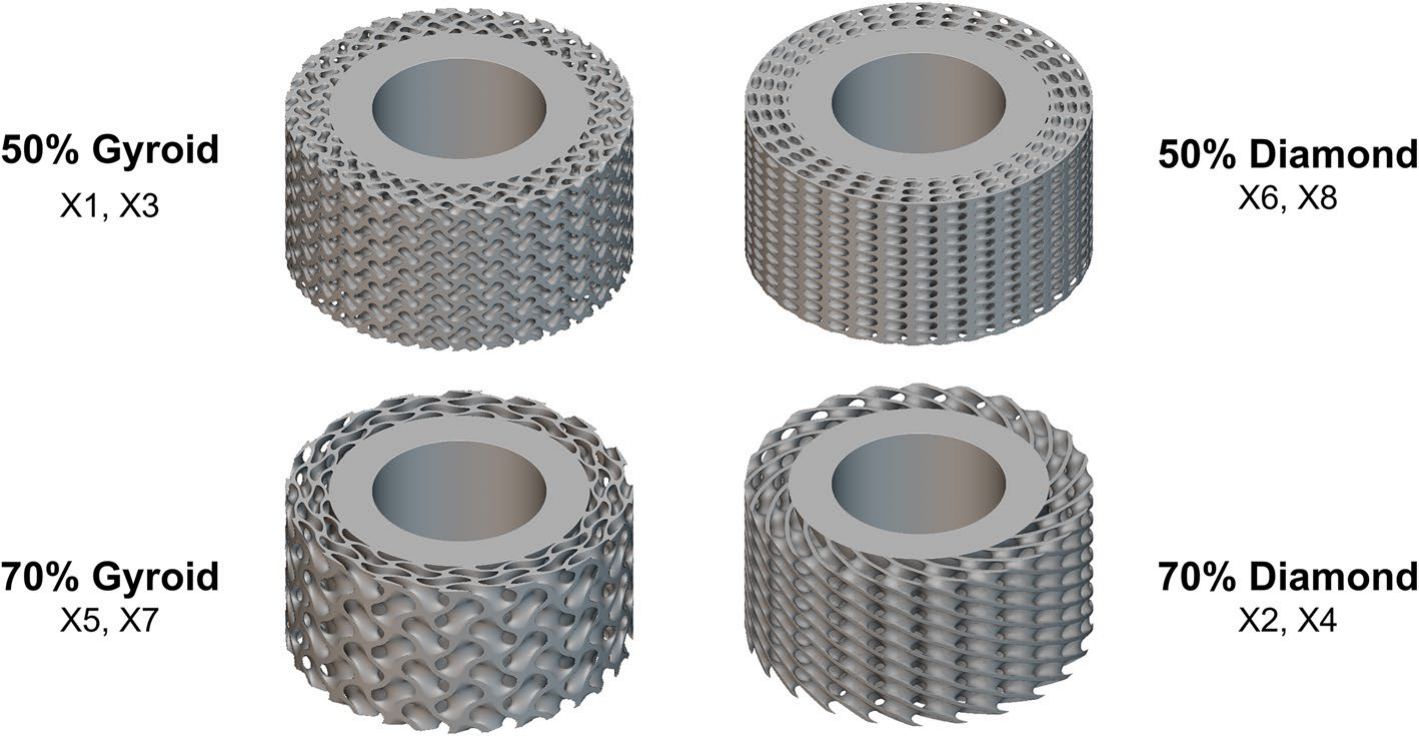
Isometric digital views of the four designs used for the Taguchi design of experiments. The percentage indicates the percent porosity and “gyroid” or “diamond” indicates the type of triply periodic minimal surface used for the porous region. The experiment number (X1, X2, X3, etc.) corresponds to the set of print parameters and levels that use that design in Table 2

The cylindrical specimens were designed using nTop 5.24.3 (nTop Inc., New York, NY) with three distinct regions: a hollow center (*r* = 10 mm), a solid region (*r* = 5 mm), and a porous region (*r* = 5 mm). The overall diameter of the part was 40 mm and the height was 20 mm. Sample dimensions and features were chosen to approximate a simplified version of a cone/sleeve for a revision total knee arthroplasty (Fig. 1). The design of the porous region used a triply period minimal surface with a thickness of 0.8 mm and either a gyroid or diamond geometry based on the Taguchi experiment. The parameters of the unit cell were adjusted to achieve either 50 or 70% porosity, also based on the Taguchi experiment (Fig. 2). STL files for each combination of triply periodic minimal surface geometry and porosity were exported from nTop and sliced using Simplify 3D 5.1.2 (Simplify 3D LLC, Cincinnati, OH).

### Additive Manufacturing of Specimens

All samples were printed on a third-generation medical fused filament fabrication (FFF) 3D printer (EXT 220 MED, 3D Systems, Inc., Rock Hill, SC). Commercially available technical grades of 1.75 mm PEEK filament (PEEK-OPTIMA™ LT1, Invibio Ltd., United Kingdom) and 1.75 mm LM PAEK (VICTREX AM200, Victrex PLC, United Kingdom) were used in this study. All filaments were dried overnight prior to use. Four samples were printed for each experiment (X1-X8) and for each material (PEEK and LM PAEK) for a total of 64 printed samples, with the average print time of each part being 2.5 hours.

### Mechanical Testing

The test order was randomized by design and specimen number prior to mechanical testing (*n* = 4). All samples were compressed on an MTS Exceed electromechanical load frame (MTS Systems, Eden Prairie, MN), fitted with 45° shear fixtures according to ASTM F2077. Each test was conducted at 10 mm/min, with a 100 N preload. Force was recorded as a function of axial displacement to determine ultimate load and stiffness for each sample. Tests were ended once the load drop exceeded 200 N after the ultimate load was achieved. Stiffness was calculated as the slope of the linear region of the force vs. displacement curve.

While ASTM F2077 is typically used for “Intervertebral Body Fusion Devices,” it was chosen for this experiment due to the relatively similar shape and loading conditions between fusion devices and these hollow cylinders. Shear loading at 45° was chosen instead of axial compression to increase the robustness of the experiment and to ensure that the load was applied to the outer porous region of the cylinders.

### Micro-computed Tomography

To determine the print fidelity and evaluate the actual printed porosity of samples, micro-computed tomography (Micro-CT) was used (Xradia 620 Versa X-ray Microscope, Zeiss, Oberkochen, Germany). The system was set to operate at a voltage of 50 kV and a power of 4 W, and a flat panel detector was used to image multiple samples at once. Image projections were collected and processed using Dragonfly 3D 2025 (Comet Technologies Inc., Montreal, Canada). Qualitatively, samples were considered to have “printed accurately” when their printed state was uniform throughout and reflected the appearance of their corresponding digital model. Volume comparison analysis was used to compare the void space with the volume of the printed material for the porous region of each sample (*n* = 1).

### Statistical Methods

The statistical analyses for the Taguchi method were computed in Minitab 22.1 (Minitab LLC, State College, PA). The SNR (Equation 1) for each experiment was calculated and then compared based on each parameter and level to determine the rank of how each parameter influenced the ultimate load. An analysis of variance (ANOVA) was also performed to evaluate which parameters had a significant impact on the performance characteristic of ultimate load (*n* = 4, *α* = 0.05). Parameter interactions were also considered. Using Cohen’s *d* for a large effect size, 0.8 [44], the minimum sample size to achieve a power of at least 0.8 for eight groups was determined to be four (1 − *β* = 0.835), calculated using G*Power (version 3.1.9.7, Heinrich-Hein- Universität, Düsseldorf, Germany) [45]. Further information regarding the mathematical analysis of the Taguchi method can be found in a previous paper [32].

For Micro-CT samples, the percent error was calculated by comparing the observed porosity of the porous region with the as-designed porosity. A Student’s t-test was used to compare the errors between PEEK and LM PAEK groups (*n* = 8, *α* = 0.05). This statistical analysis was performed in GraphPad Prism (version 8, GraphPad Software Inc., Boston, MA).

## Results

### Mechanical Analysis

**Fig. 3:**
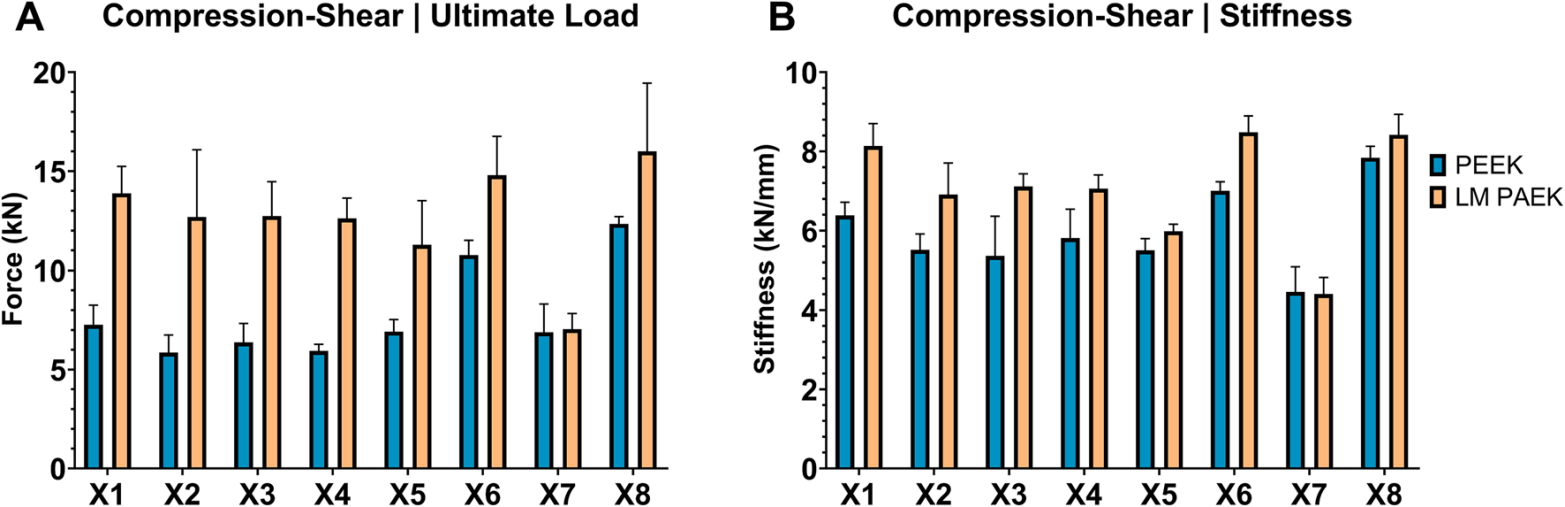
Mean and standard deviations for ultimate load (**A**) and stiffness (**B**) for experiments X1-X8 for PEEK (blue) and LM PAEK (orange) (*n* = 4)

The ultimate load results in 45° shear showed that LM PAEK X8 withstood the largest load at 16.0 ± 3.4 kN, followed by LM PAEK X6 at 14.8 ± 2.0 kN (Fig. 3A). Every LM PAEK group mean exceeded the corresponding PEEK group, with the smallest difference observed in the X7 groups (LM PAEK: 7.04 kN vs. PEEK: 6.88 kN). The best performing PEEK groups were X8 at 12.34 ± 0.36 kN, followed by X6 at 10.76 ± 0.74 kN.

Stiffness values were also calculated for each sample (Fig. 3B), with LM PAEK having stiffer samples than PEEK for each group except X7 (LM PAEK: 4.41 ± 0.42 kN/mm vs. PEEK: 4.46 ± 0.63 kN/mm). The raw plots for force vs. displacement for PEEK and LM PAEK samples are available in the supplemental section (Figs. S1, S2).

### Taguchi Optimization

**Fig. 4:**
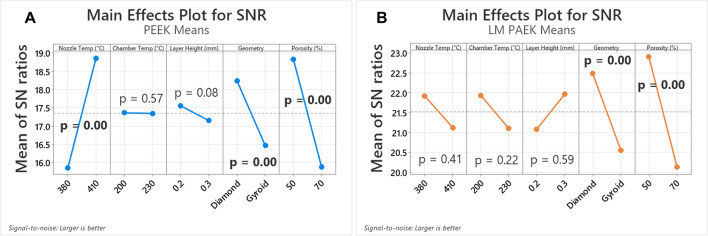
Main effects plots for signal-to-noise ratio means of PEEK (**A**) and LM PAEK (**B**). The larger of the two SNR means indicate which level of each parameter resulted in higher ultimate loads. The p-values for each parameter are shown with statistically significant p-values shown in bold

SNR values were calculated for each parameter level and compared based on ultimate load results. The ranked order of most to least impactful parameter for PEEK was nozzle temperature (*F* = 81.01, *p* = 0.000), porosity (*F* = 76.22, *p* = 0.000), geometry (*F* = 34.31, *p* = 0.000), layer height (*F* = 3.40, *p* = 0.077), and chamber temperature (*F* = 0.33, *p* = 0.569) (Fig. 4A). The ranked order for most to least impactful parameter for LM PAEK was porosity (*F* = 16.83, *p* = 0.000), geometry (*F* = 11.05, *p* = 0.003), chamber temperature (*F* = 1.60, *p* = 0.217), nozzle temperature (*F* = 0.69, *p* = 0.414), and layer height (*F* = 0.29, *p* = 0.594) (Fig. 4B).

Based on the means of the SNR values obtained from the Taguchi analysis, the optimal print parameters for each material are shown in Table 3. Interaction plots for the various parameters for both PEEK and LM PAEK samples are available in the supplemental, with the optimal print parameters changing depending on the combination of geometry and temperatures chosen (Fig. S3).

**Table 3:** Optimal print parameters for producing the strongest partially porous cylinder for PEEK and LM PAEK based on the Taguchi analysis performed

Varied parameters		PEEK	LM PAEK
P1	Nozzle Temp. (°C)	410	380
P2	Chamber Temp. (°C)	230	200
P3	Layer Height (mm)	0.2	0.3
P4	Geometry Type	Diamond	Diamond
P5	Porosity (%)	50	50

### Micro-CT

**Fig. 5:**
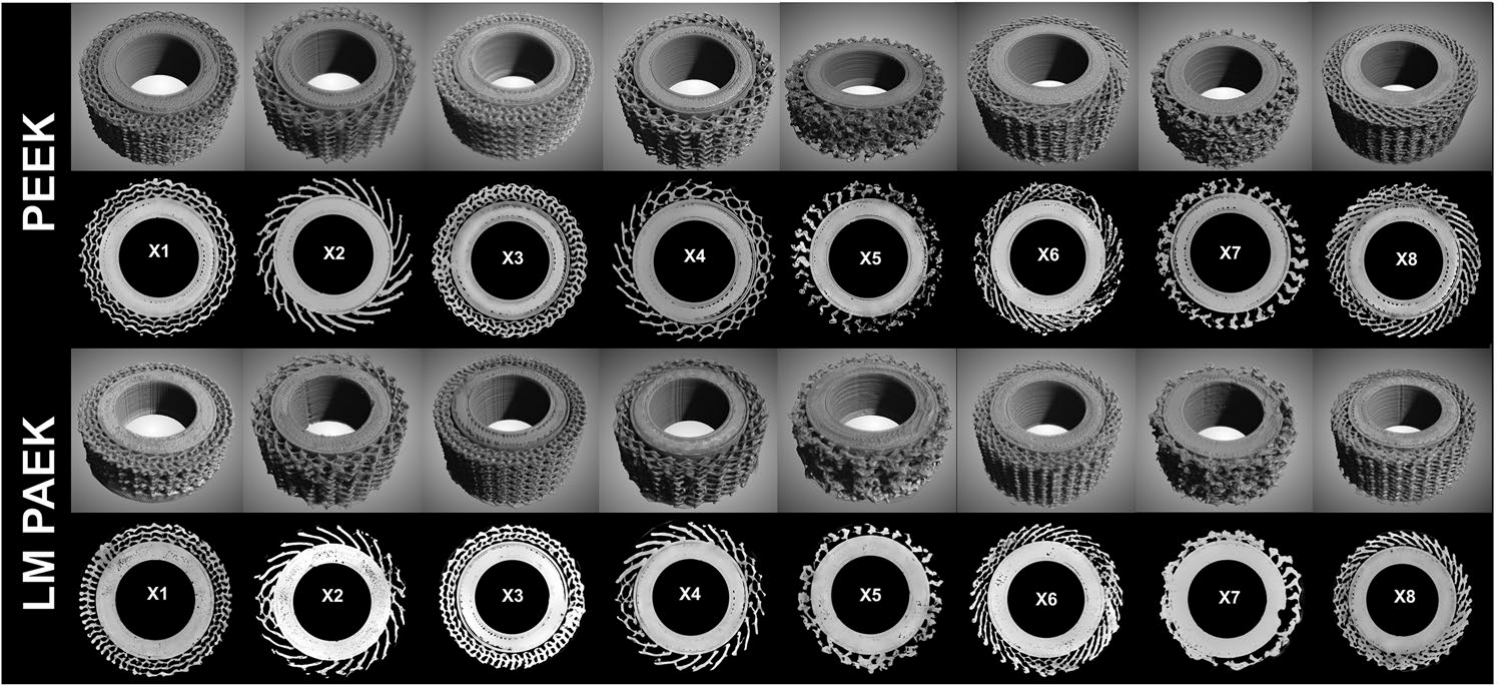
Micro-CT images showing the print quality of cylinders printed in PEEK (row 1 and 2) and LM PAEK (row 3 and 4) for each experiment (X1 in column 1, X2 in column 2, etc.)

Micro-CT images of a representative sample from each group, along with their cross-sectional view are shown in Fig. 5. Qualitatively, the unique porous networks appeared accurate to the digital model, except X5 and X7 for both materials. Both X5 and X7 were 70% gyroid, suggesting this design was more difficult to print with the parameters tested in this Taguchi analysis.

The comparison between the observed porosities of the porous regions and the as-designed porosities of each model is shown in Table 4. Experiment X1 with 50% gyroid, had the lowest percent error for both materials, while X3, also with 50% gyroid, had the highest error for both materials. Although the porous regions of X5 and X7 appeared messy for both materials, the measured porosities of the overall regions were within ~ 10% error.

**Table 4:** Measurement of percent porosity based on Micro-CT analysis (*n* = 1)

Experiment #	Expected porosity (%)	Measured porosity			
		PEEK (%)	PEEK error	LM PAEK (%)	LM PAEK error
X1	50	50.9	1.8%	48.8	-2.4%
X2	70	64.6	-7.7%	61.4	-12.3%
X3	50	40.0	-20%	37.5	-25%
X4	70	62.1	-11.3%	64.0	-8.6%
X5	70	64.8	-7.4%	65.4	-6.6%
X6	50	46.6	-6.8%	45.4	-9.2%
X7	70	64.5	-7.9%	62.9	-10.1%
X8	50	45.8	-8.4%	38.1	-23.8%
		Mean ± Std:	-8.46 ± 6.0%	—	-12.25 ± 8.0%

## Discussion

PEEK is known for its biologically inert surface and is approved for use in several approved medical devices [46]. However, parts made from this material are typically manufactured via injection molding or machined from stock rods. The material used in this experiment is made from the same stock used for other PEEK devices, but in the form of a 1.75 mm filament. No additives are used or considerations made to optimize the material for use in FDM printing. Basgul et al (2018) reported that under compressive-shear loading, their best performing PEEK 3D printed cage reached 71% of the ultimate load that a machined cage achieved (~2.1 kN) and was significantly weaker in this aspect (*p* < 0.05) [47]. In contrast, LM PAEKs such as AM200 are optimized with additive manufacturing in mind and can be printed at slightly lower temperatures [20]. Yi et al (2021) found that LM PAEK parts were significantly stronger than PEEK parts (Victrex 151G grade) when printed in the Z direction. However, this trend was not observed in other orientations or required post-processing such as annealing to cause the difference to be significant. This difference was attributed to LM PAEK’s slow crystallizing nature, leading to increased interlayer adhesion, therefore promoting higher Z strength [20].

While the proposed clinical application being considered in this study is as a cone or sleeve in a revision knee system, there is limited data in the literature specifically addressing the forces experienced by these components. Instead, much of the data addresses how much overall force the knee experiences using implants with integrated measurement devices. A “worst-case” can therefore be established where all the load experienced by a TKA implant can be imagined to be experienced by just the metaphyseal sleeve component. One study found that axial tibial forces experienced during stair climbing could reach 3X the body weight (BW), whereas the peak anterior shear forces were much lower than the axial forces at 0.26X BW during the same activity [48]. Assuming a worst-case scenario where the axial forces were instead experienced in shear, and considering a patient profile of a 90% male aged 50–59 weighing 120 kg [49], the estimated force experienced during stair climbing based on the previous study would be 3528 N. Another telemetry study which standardized recorded data to an “average” body weight of 74.7 kg (AVER75) and used a “high” body weight of 100 kg (HIGH100), found that the greatest axial force was experienced by the implant when jogging at 6 km/h, with forces experienced during sitting down as the next highest. When selecting for the peak values of all load components experienced by a number of patients, an extreme value was developed for a patient with a high body weight (EXTREME100), which was determined to be 5551 N when jogging and 4787 N when descending stairs [50]. The worst performing group tested in our experiment was PEEK X2 with an average ultimate load of 5854 N and the best performing was LM PAEK X8 at 16000 N, suggesting that these 3D printed parts can withstand the forces experienced by a TKA implant. However, the strength of the component may also change when the design is truly cone-shaped as opposed to cylindrical.

The results of the Taguchi analysis interestingly found that the optimal print parameters for PEEK were different than the optimal parameters for LM PAEK, with PEEK preferring higher nozzle and chamber temperatures, while LM PAEK preferred lower temperatures. These findings emphasize the importance of optimizing print parameters per material. While PEEK and LM PAEK are both high-temperature semicrystalline polymers used in 3D printing, their unique chemical structures and resulting crystallization kinetics make their optimal print parameters specific to their formulation.

Based on the Micro-CT results, while the 70% diamond samples printed as expected, it was challenging to achieve an accurate print of the 70% gyroid samples. This could suggest that for the range of print parameters tested, none were optimal for such a complex geometry. This is consistent with reports in the literature, that diamond is a “simpler” triply periodic minimal surface structure than “gyroid” [41] and therefore may allow for a more lenient optimization process to produce a part that passes visual inspection. The mechanical performance outcomes for groups X5 and X7 showed that even though the porous networks appeared “messy,” the parts themselves were still relatively strong, and the porous region was well attached to the solid region of the part. The large differences in percent error for groups X1 and X3 for both materials, despite both having 50% gyroid designs, illustrate how important print parameter optimization is to achieve a quality part. If the parameters for X3 were considered optimum for most user applications, they might discard the 50% gyroid design for being “unprintable,” when in reality the design is sufficient, and instead it is the print parameters that need to be altered.

Limitations for this work include the differences in geometry between an actual metaphyseal cone or sleeve and the cylinders tested. While the shapes of the parts are similar, mechanically testing a cone poses a number of challenges that are mitigated by printing a more uniformly shaped cylinder. Care was taken to still include the different regions (hollow, solid, and porous) of the proposed device. In addition, the number of samples and types of mechanical testing that could be performed were limited by how resource-intensive it was to print each part. Future work involving the mechanical performance of non-metal metaphyseal cones would need to consider fatigue using cyclic testing. It is also important to note the LM PAEK used in this experiment, unlike the PEEK, has not yet been cleared for long-term implant applications.

## Conclusion

The purpose of this study was to optimally print a complex geometry consisting of connected hollow, solid, and porous sections out of two medically relevant 3D printable PAEK (PEEK and LM PAEK) materials. Taguchi optimization successfully allowed for the variation of multiple parameters, while reducing the amount of time and materials necessary to print specimens for testing. We found that LM PAEK specimens exhibited higher stiffness and strength than PEEK specimens, in 45° shear, but all designs, irrespective of PAEK selection, surpassed a theoretical worst-case scenario of ~ 5.5 kN. Further refinement of the geometry would be needed to closely replicate the design of a metaphyseal cone or sleeve, but the Micro-CT results confirm that highly complex porous designs are possible at this scale without compromising on strength.

## Supplementary Information

Below is the link to the electronic supplementary material.

Supplementary file1 (TIF 404 kb)

Supplementary file2 (TIF 376 kb)

Supplementary file3 (TIF 929 kb)

## Data Availability

The data that support the findings of this study are available from the corresponding author upon reasonable request.
