# Supplementary figures and images for: Additively Manufactured Partially Porous PAEK Topologies for Proximal Tibia Revision Cones and Sleeves
Source: Ann Biomed Eng. 2026 Feb 19;54(7):2337–45. doi: 10.1007/s10439-026-04042-8 (PMC13290881; doi:10.1007/s10439-026-04042-8)

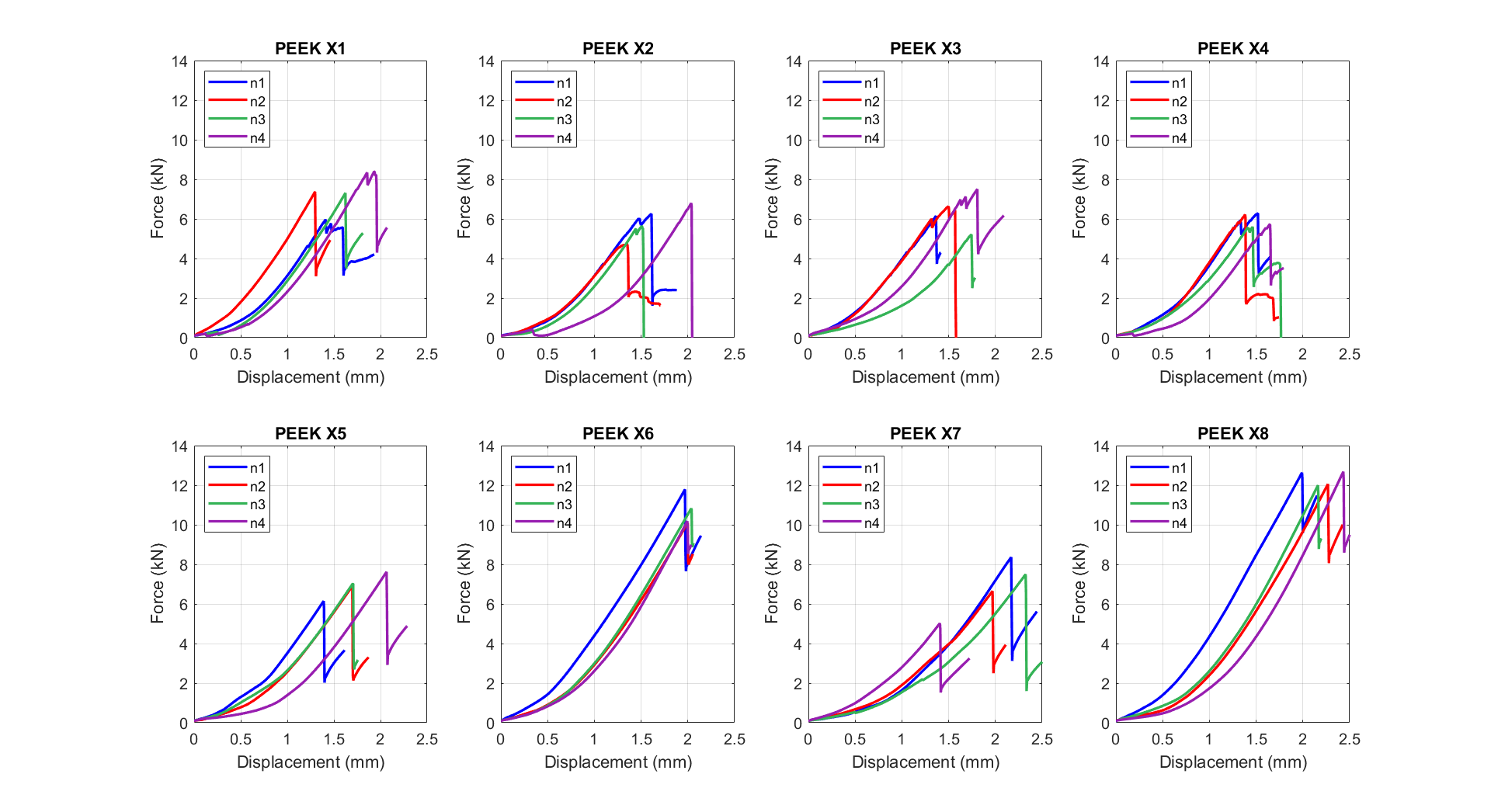

Supplement: Supplementary file 1 — Supplementary file1 (TIF 404 kb) [file 10439_2026_4042_MOESM1_ESM.tif]

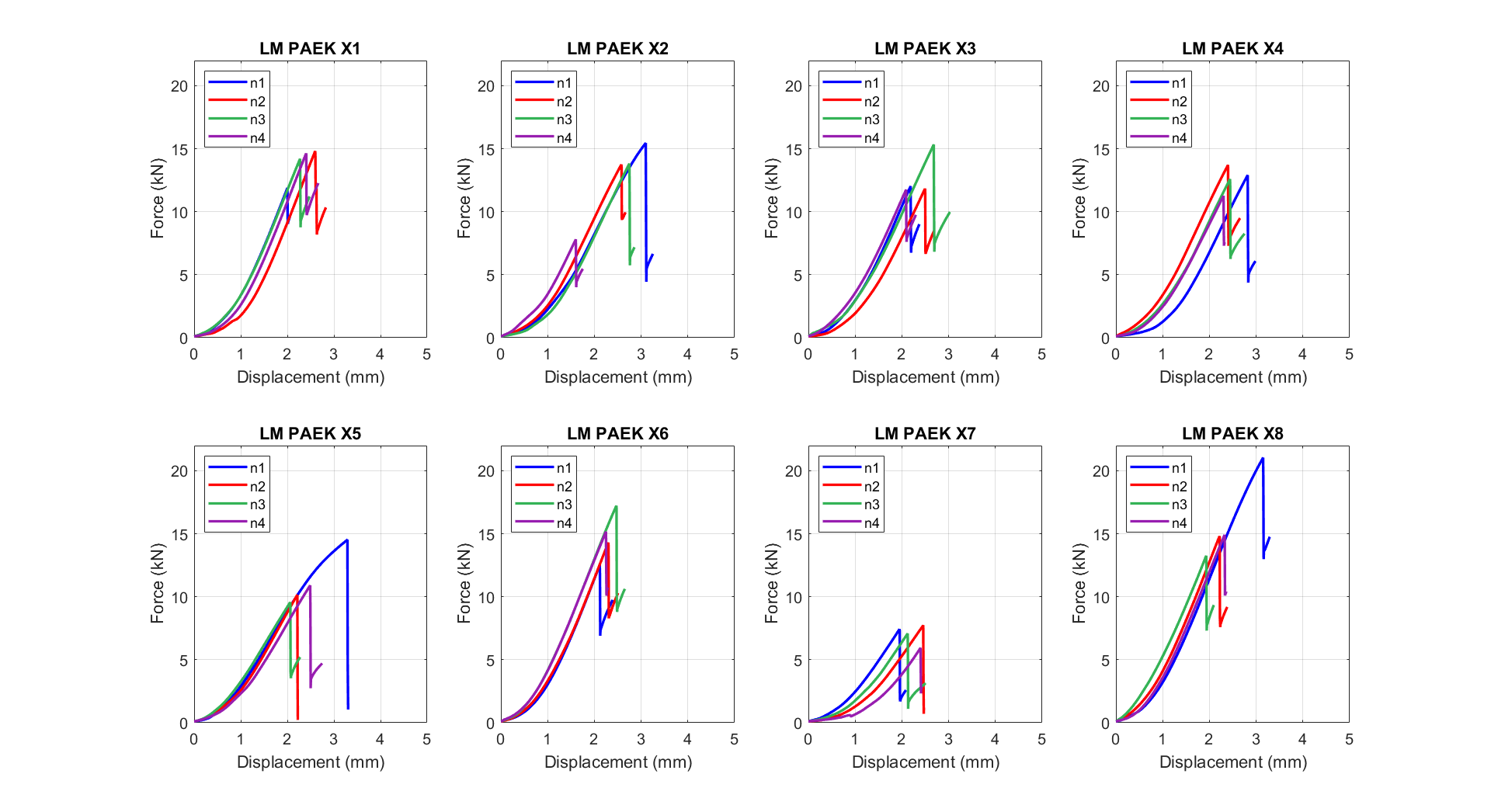

Supplement: Supplementary file 2 — Supplementary file2 (TIF 376 kb) [file 10439_2026_4042_MOESM2_ESM.tif]

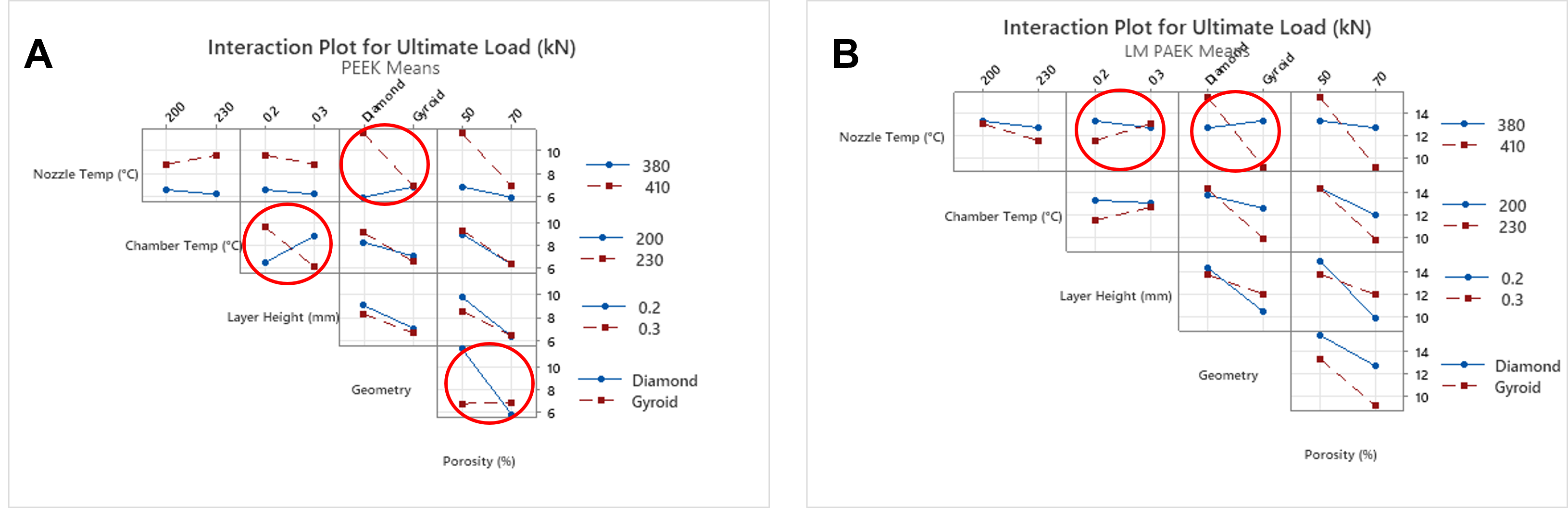

Supplement: Supplementary file 3 — Supplementary file3 (TIF 929 kb) [file 10439_2026_4042_MOESM3_ESM.tif]
